# Efficacy of methanolic extracts of some medicinal plants on wound healing in diabetic rats

**DOI:** 10.1016/j.heliyon.2022.e10071

**Published:** 2022-08-02

**Authors:** Ahmad Z. Alsarayreh, Sawsan A. Oran, Jumah M. Shakhanbeh, Khaled M. Khleifat, Yaseen T. Al Qaisi, Ibrahim I. Alfarrayeh, Ayah M. Alkaramseh

**Affiliations:** a Department of Biological Sciences, Faculty of Sciences, Mutah University, Al-Karak, Jordan; b Department of Biological Sciences, Faculty of Sciences, University of Jordan, Amman, Jordan; c Faculty of Allied Medical Sciences, Al-Ahliyya Amman University, Amman, Jordan

**Keywords:** Cytotoxicity, Diabetes, Medicinal plants, Wounds, Wound healing

## Abstract

**Background:**

One of the primary health concerns for diabetes individuals is wounds. The used drugs have several side effects, urging the need for new natural sources for therapeutics.

**Materials and methods:**

This study was designed to estimate the wound healing potential of the methanolic extract of *Globularia arabica* and *Malva sylvestris* leaves and *Rhus coriaria* fruits. plant extracts were orally administered to the rats to determine their effect on the wound-healing process.

**Results:**

Plant extracts significantly increased the contraction of the wound in non-diabetic and diabetic rats (P < 0.05) and increased the fibroblast's proliferation and migration resulting in a faster healing process. The plant extracts have no cytotoxic effects. The proliferation assay exhibited the lowest cell mortality after treatment with plant extract.

**Conclusion:**

These findings may indicate that the methanolic leaf extract of the above plants can be used as new therapeutics for wound healing in diabetic patients.

## 1 Introduction

Plants offer many advantages of being a source of medicinal remedies, since they are easily available in the local surroundings, have no side effects, are cost-effective, and are easy to use [1, 2]. Medicinal plants in Jordan are around 363 species that are used in traditional medicine and have many pharmacological effects [3].

Diabetes is a severe disease and one of the world's most common chronic diseases. It affects about 381 million adult populations globally [4], moreover, it is estimated that about 175 million cases are still undiagnosed [5]. The disease is becoming increasingly prevalent; for example, in the united states, 11 million people were diagnosed in 2001, accounting for about 4% of the whole population, number is expected to increase to 29 million during 2050 [6]. International Diabetes Federation (IDF) estimates that these numbers will continue to grow up in the future [7]. Type two diabetes is the most public, secretarial for 85–90 % of diabetic patients, whereas type 1 diabetes accounts for about 10–15 % only [8].

**Figure 1:**
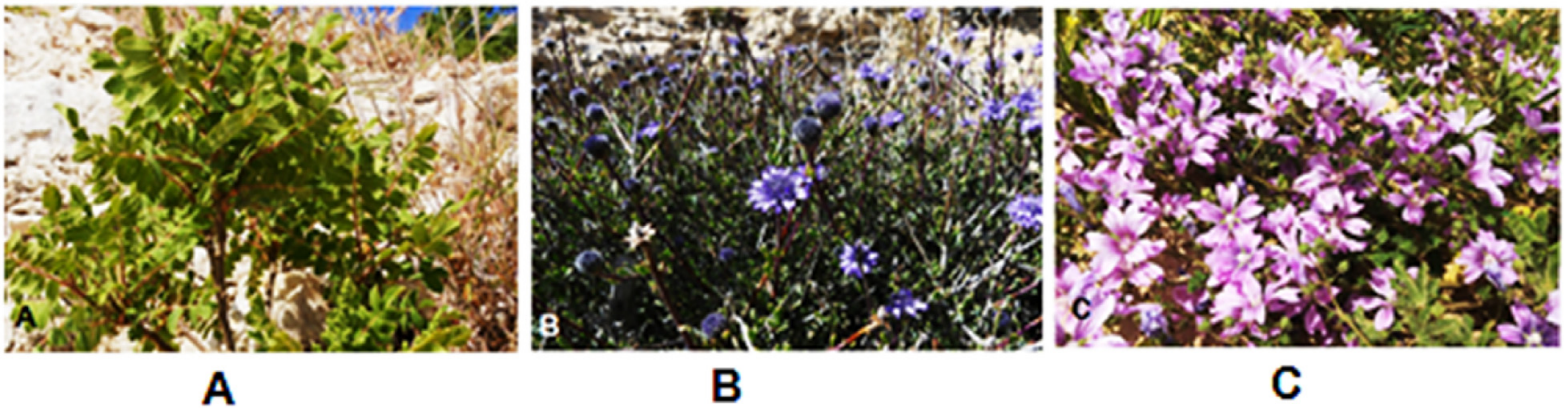
Representative photos for the plant species investigated in this study: *Rhus coriaria* (A), *Globularia arabica* (B), and *Malva sylvestris* (C).

The present study is undertaken to provide a scientific approach to the effectiveness of selected medicinal plants in the management of injuries in diabetic patients. The basic criterion for the selection of these plants was their use of these plants in traditional medicine. Most importantly, a huge number of these plants are being used by the ancestral communities for the handling of injuries, cuts, and skin diseases. Therefore, three plant species were selected to be evaluated for their potential healing activity of wounds *in vivo* and *in vitro.* Those plants are *Rhus coriaria, Globularia arabica*, and *Malva sylvestris* (Figure 1).

*R. coriaria* (Figure 1A) with the common name “Sumac”. It is a very general flavor and the main turning agent [9]. Numerous pharmacological properties, including antimicrobial, hepatoprotective, hypoglycemic, antioxidant, DNA-protective, and antibacterial, have been demonstrated [10]. In Jordan, people use it in folk medicine as an anti-inflammatory agent [11], and as a traditional spice because of its tart flavor [12]. *Rhus* is also considered a good source of tannins with hydrolyzable groups, Gallo tannins [13]. Moreover, it was previously reported that *R. coriaria* has high potential activity in wound healing [14].

*G. arabica* (Figure 1B), which belongs to the family Plantaginaceae, is the only species of *Globularia* that grows in Jordan [15, 16]. It is commonly used by Jordanian people in traditional medicine due to its known antimicrobial and anti-tumorous activities [3, 12]. To treat a wound, the leaves of *G. arabica* are pounded into a powder or chewed, then dusted or spread over the wound [17]. According to Rodríguez-Pérez et al [18], The methanolic extract of *Globularia* spp enhances burn wound healing and inflammation in rats and has antibacterial and antioxidant properties.

*M. sylvestris* (Figure 1C) belongs to the family Malvaceae and it is commonly known as Mallow [19]. Its therapeutic characteristics have been described as anti-inflammatory, anticancer, and having a good effect on gingivitis, abscesses, tooth discomfort, urological illness, insect bites, ulcerous wounds, as well as particular disorders affecting several body systems [20]. Young leaves are consumed raw in salads, while older leaves and shoots are utilized in soups and as boiled vegetables [19]. *M. sylvestris* has anti-inflammatory qualities due to the presence of compounds such as mucilage, flavonoids, and tannins [21]. According to several past studies, the flowers of *M. sylvestris* are used to treat cut wounds, cutaneous infected wounds, dermatitis, and inflammatory disorders like stomach disorders and inflammation of the lining bronchial tubes [22]. Moreover, the chloroform flower extract of *M. sylvestris* gave a good healing potential in diabetic rats [23].

In this study, we aimed to evaluate the wound-healing potential of methanolic extracts of *G. arabica* and *M. sylvestris* leaves, and *R. coriaria* fruits in diabetic rats, as well as the cytotoxic effect of these plant extracts on a human fibroblast cell line.

## 2 Methods

### 2.1 Plant collection and identification

In July 2020, leaves of *G. arabica*, *M. sylvestris* (estimated age 3 months), and fruits of *R. coriaria* (2 months after fruits formation) were collected from various locations in Jordan. Prof. Sawsan Oran, Department of Biological Sciences, University of Jordan, Amman, Jordan, authenticated the plants. The University of Jordan's Herbarium received voucher specimens (Mu2021-22,2 and 24) [24].

### 2.2 Preparation of the methanolic extract

*M. sylvestris* and *G. arabica* leaves, and *R. coriaria* fruits were cleaned, dried, and ground in a blender before being immersed in 100% methanol. Dried leaves were immersed in methanol (1:10 w/v ratio) for three days at room temperature with constant shaking [25]. The solution was filtered through a Whatman filter paper to remove the solid impurities and then dried using a rotary evaporator set to 50 °C under low pressure [26]. The crude extracts were stored in airtight containers at -20 °C.

### 2.3 Animals

Male rats (180–200 g) were used to test the healing activities and anti-inflammatory of methanolic extracts of *G. arabica*, *M. sylvestris* leaf, and fruits of *R. coriaria*. Investigators picked male rats because they believe that hormonal fluctuations during the female reproductive cycle caused female rats to behave differently to the identical stimuli, rendering them unpredictable and providing scientists with varying results in repeated studies. The animals were kept in separate standard plastic cages in the animal house at Mutah University, Jordan. The temperature was kept at 24 °C by alternating between 12 h of light and 12 h of darkness to maintain the biological clock of the animal as it was in its natural environment so that the results of the tests would be reasonable. Jordan University's ethical committee granted the study ethics under reference number 47–2021. Before conducting the experiments, the animals were given time to adjust to their new surroundings. During the experiments, the animals were separated using separate cages. Various methods were used to collect blood samples (Figure 3). Biochemical tests were also done on the serum. The doses of plant extracts given were chosen based on a previously reported LD_50_ in rats (*R. coriaria* fruit extract of more than 1975 mg/kg body weight [27], *G. arabica* was up to 1000 mg/kg wt [28], and *M. sylvestris* was up to 2000 mg/kg wt [29]).

### 2.4 Induction of diabetes

A single dose intraperitoneal injection of streptozotocin (65 mg/kg body weight) produced diabetes. Diabetes was established after three days by measuring blood glucose concentration in the tail vein with glucometer strips (On-call plus, Hanover, Germany, Mutah University) [30]. Animals with blood glucose level more than 200 mg/dL were considered diabetic [31].

### 2.5 Plant extract toxicity study

The present study used male rats (180–200 g) that had been fasted overnight. The animals have been separated into five groups each group consisting of two rats. Groups 1–4 were given 2 mL of plant extract orally, as shown in Table 1. The same route was used for the control (group 5) to receive normal saline (2 mL/group). This method has three stages, with the results of each indicating whether testing should be stopped or continued to the next stage. Within 24 h, general toxicity symptoms and mortality were observed in each group. If there is no death documented at these stages, the extract substance LD_50_ is assumed larger than 5000 mg/kg body weight, representing a great degree of safety. If death is recorded at a certain dose in any of the stages, Another test should be made to verify if the extract caused death. This test simply involves administering the dose of extract that caused death (or low dose that caused death in a condition where more than one death was recorded) to four animals. The rats should then be detected for 1 h after treatment and every 2 h for the next 24 h. If at least two of the four animals die, this should serve as verification and authentication of the test results. This method has several advantages, including the use of fewer animals, the investigation of a varied sort of dosages, and the fact that it is inexpensive [32].

**Table 1:** Doses for the toxicity study.

Step	Doses (mg/kg bw)				
	G 1	G 2	G 3	G 4	G 5
1	100	200	400	800	Saline
2	1000	1500	2000		
3	3000	4000	5000		

LD_50_ was calculated according to the following formula [32].

LD_50_ = (M0 + M1) / 2

Where M0 is the highest dose of plant extract that gave no death, and M1 is the lowest dose of plant extract that gave death.

**Figure 2:**
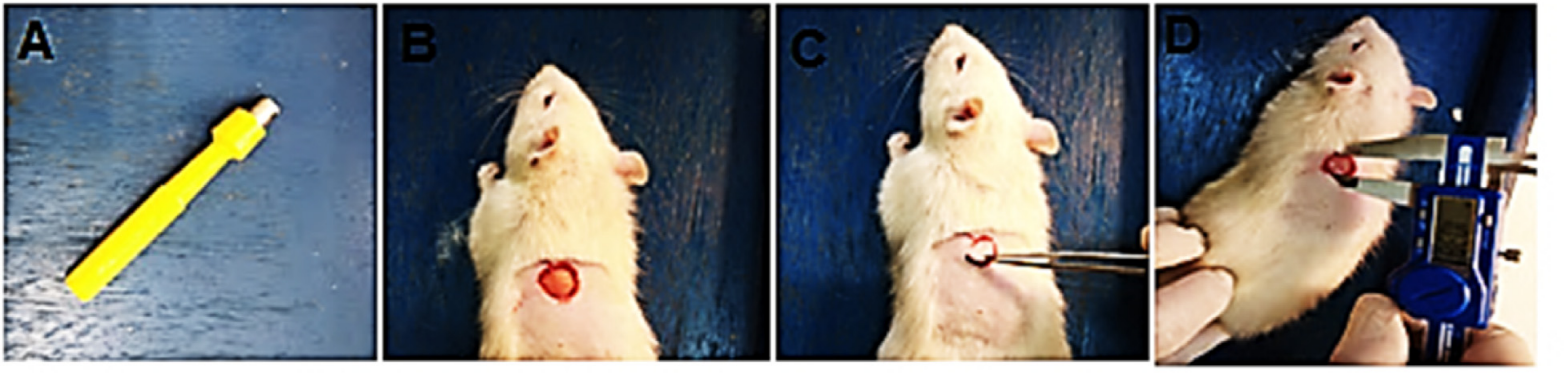
Wound creation in rats. A. 10-mm diameter biopsy punch. B–C. Skin removal. D. Wound Measurement [27].

Diethyl ether was inhaled to anesthetize rats. The dorsal aspect was shaved with an electrical clipper (Geemy professional hair clipper), and the skin was removed with a 10 mm biopsy needle (Disposable Biopsy Punch, ROBBINS Instrument, Mutah University) Figure (2) [27]. Contraction, which is primarily responsible for wound closure, was investigated until the wounds were completely epithelized. The percentage of contraction was calculated according to the formula:

$$\text{Contraction ​\%}=\left(\frac{\text{Original ​wound ​diameter ​}-\text{ ​exact ​day ​wound ​diameter}}{\text{Original ​wound ​diameter ​ ​}}\right)\times 100$$

**Figure 3:**
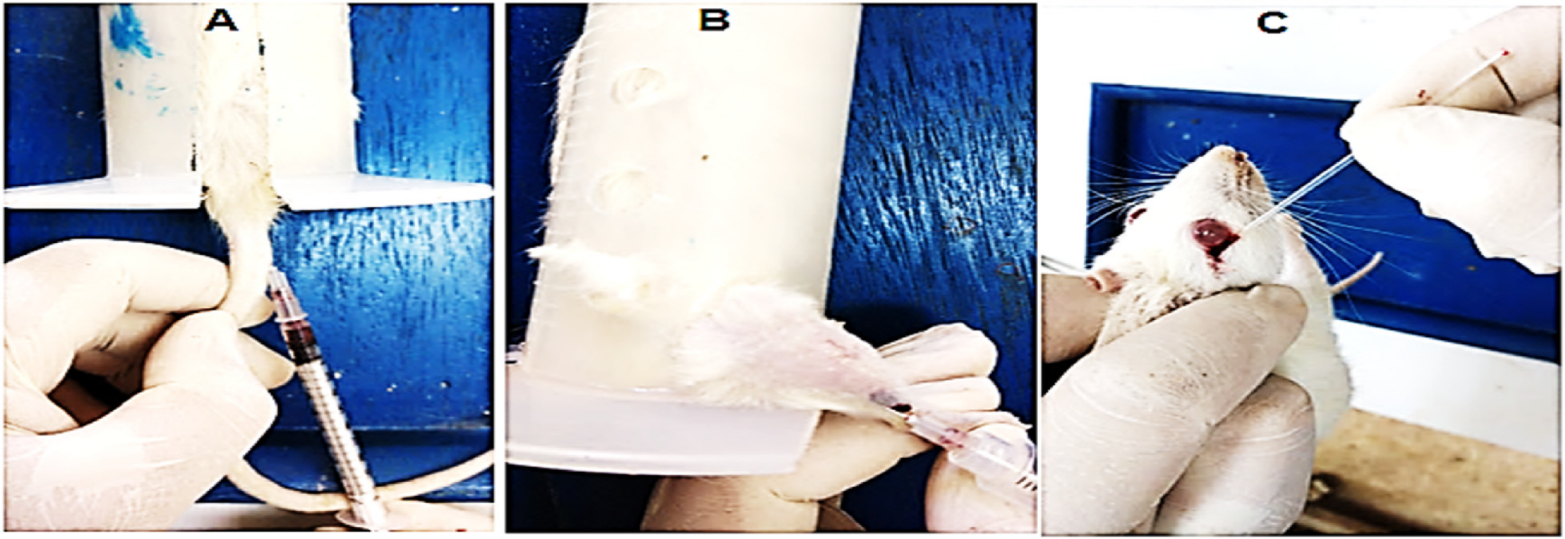
Blood collection methods. A: Tail vein B: Lateral Saphenous Vein C: Retro Orbital Sinus Vein [27].

The period of epithelization was observed by noting the number of days required for the eschar to fade away, leaving no raw wound behind [33]. Blood samples were collected as shown in figure (3) [27].

### 2.6 Experiment design

Animals were separated into two main groups non-diabetic (1–3) and diabetic (4–6) (Table 2). Each main group is composed of three subgroups of five animals each (n = 5) as follows: Group 1(Control): received vehicle (0.5% sodium carboxymethyl cellulose suspension in normal saline); Group 2 (Positive control): received Vitamin E (100 mg/kg bw); Group 3: received plant extract (200 mg/kg bw) extract in the vehicle. All drugs and plant extract were administered at equivalent volumes of 5 ml/kg bw for 15 days. The wounds were created as described by Mieczkowski et al. [34]. Wound areas were measured using a caliper on days zero, three, six, nine, twelve, and fifteen. The wounds were pictured using a digital camera. The contraction of the wounds was studied until day 15.

**Table 2:** Treatment groups in orally administrated methanolic plant extracts.

Non-diabetic rats		Diabetic rats	
Group	Treatment	Group	Treatment
1	Normal saline	4	Normal saline
2	Vit.E (100 mg/kg)	5	Vit. E (100 mg/kg)
3	200 mg/kg plant extract	6	200 mg/kg plant extract

### 2.7 Fibroblast proliferation and migration in wound healing activity of methanolic plant extract *in vitro* study

#### 2.7.1 *In vitro* cytotoxicity test

Plant extracts were tested for cytotoxicity against a fibroblast cell line that was kindly gifted by Prof. Yaser Bustanji's lab at the University of Jordan. The cells were grown in Dulbecco's Modified Eagle Medium. Then it was cultivated in 5% CO_2_ at 37 °C. After 3–4 days, cells were fractionated by removing the culture liquid, detaching the cells with 2 mL of trypsin, then adding fresh medium [35]. The plant extract was tested with different concentrations (10–100 μg/mL). All experiments were carried out in triplicates. The dose-response curve was used to calculate the IC_50_. The cytotoxicity of the plant extracts was determined with different concentrations of plant extracts (10, 25, 50, and 100 μg/mL) using a 24-well. Changes in cell shape and morphology were regarded as cytotoxic indicators. As a negative control, cell lines without extract were used [36].

#### 2.7.2 Antiproliferative assay

Giemsa staining method was used to measure the inhibition of cell proliferation after the tested compounds were applied to cell lines. The media was aspirated from the wells, then the wells were washed with 0.5 mL PBS and fixed with methanol for 9 min at 36 °C. After that, the plates were dried for 2 min. Each well was treated with Giemsa stain (1:10 in PBS) for 9 min. Then rinsed with deionized water after aspirating the dye. Using 0.1N HCl, the bounded stain was extracted, and antiproliferative activity was determined using an enzyme-linked immunosorbent assay (ELISA) microplate reader at 630 nm (ELX 800 Instrument). Cell mortality was represented as the percentage of living cells in the tested sample compared to the control [37]. The percentage of mortality was calculated using the following formula [38].

$$\text{Mortality ​}\left(\text{\%}\right)=100-\left(\frac{\text{Absorbance ​of ​control}-\text{Absorbance ​of ​treated ​cells}}{\text{Absrobance ​of ​control}}\times 100\right)$$

#### 2.7.3 Migration assay

At a density of 35,000 cells per well, fibroblasts were seeded and incubated until confluent. Using a 200 μl pipette tip as described by Juszczak et al [39], a scratch was made in each well. Before adding the medium conditioned with different concentrations of each plant extract (10 and 20 μg/ml), the media was withdrawn and the cells were rinsed with PBS. After 0, 12, 24, and 48 h of induced damage, different regions along the scratches of each well were examined using optical microscopy. Using Image J, the distance between the scratch's edges was measured and expressed as a percentage of area closure relative to untreated control cells [40].

### 2.8 Statistical analysis

The data were presented as means ± standard deviations. The statistical significance of differences between groups was determined using Graph Pad Prism version 7, P ˂ 0.05 was considered significant.

## 3 Results and discussion

### 3.1 Toxicity study

Lethal doses of the extracts of *R. coriaria, G*. *arabica,* and *M. sylvestris* were found to be 4500, 3500, and 4500 mg/kg bw, respectively. Previous studies of acute toxicity have revealed that all the doses of *R. coriaria* and *M. sylvestris* extracts were safe and non-toxic up to 4500 mg/kg bw [27, 29], and *G*. *arabica* methanolic extract was safe up to 3500 mg/kg bw [41].

### 3.2 Effect of orally administrated plants extracts on cutaneous wound in diabetic rats

**Figure 4:**
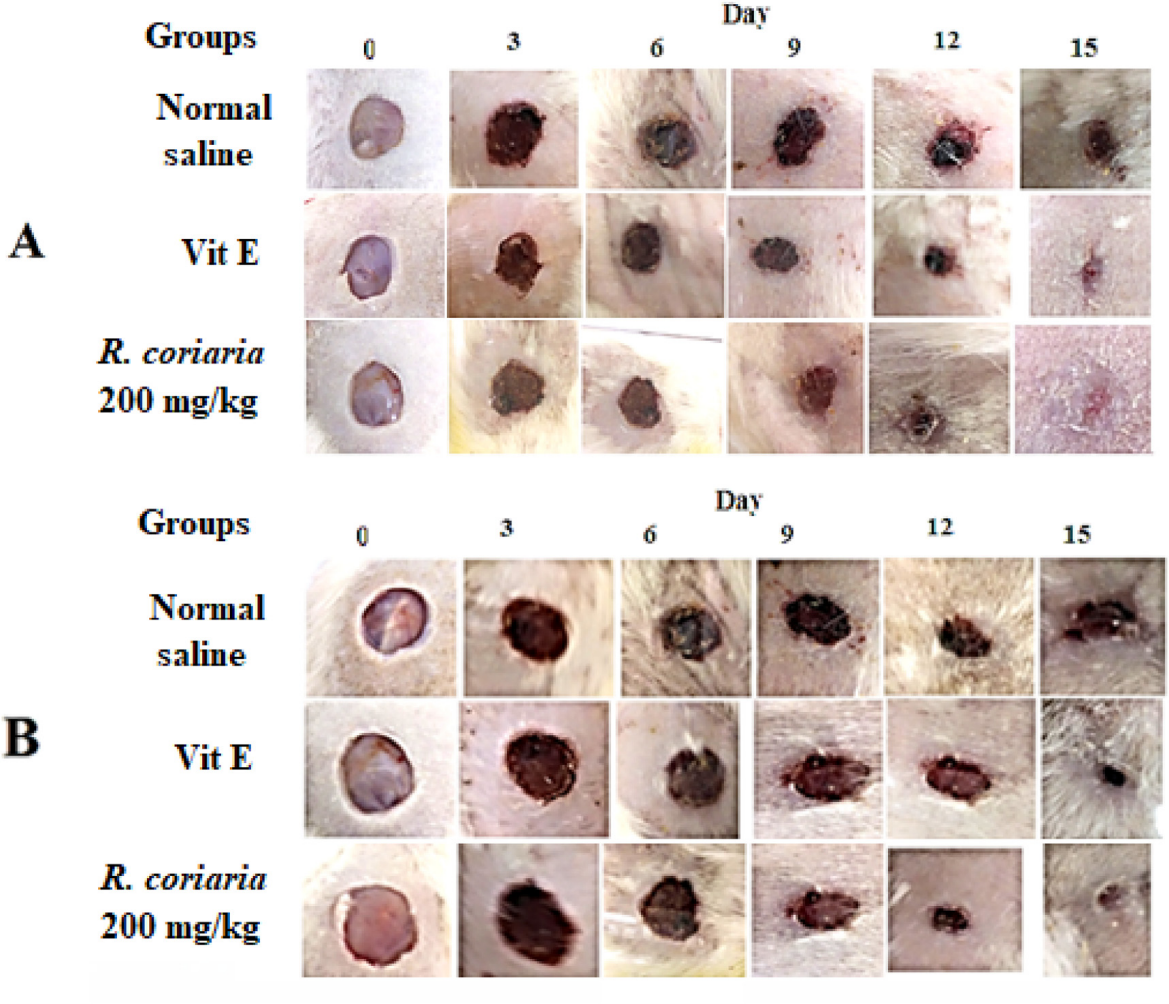
Morphological representations of wound contraction treated orally with *R. coriaria* methanolic extracts in non-diabetic (A) and diabetic (B) rats.

**Figure 5:**
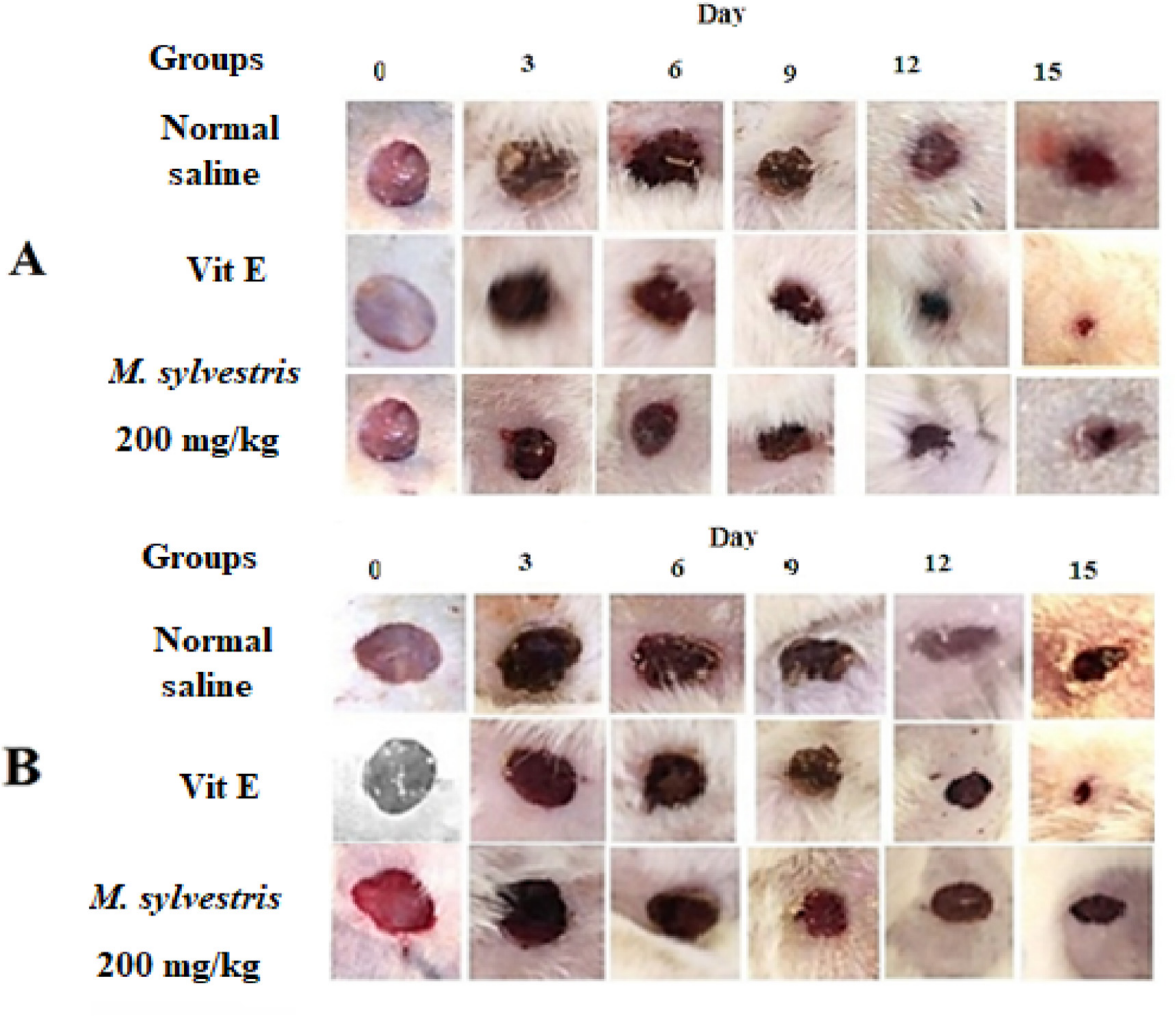
Morphological representations of wound contraction treated orally with *M. sylvestris* methanolic extracts in non-diabetic (A) and diabetic (B) rats.

**Figure 6:**
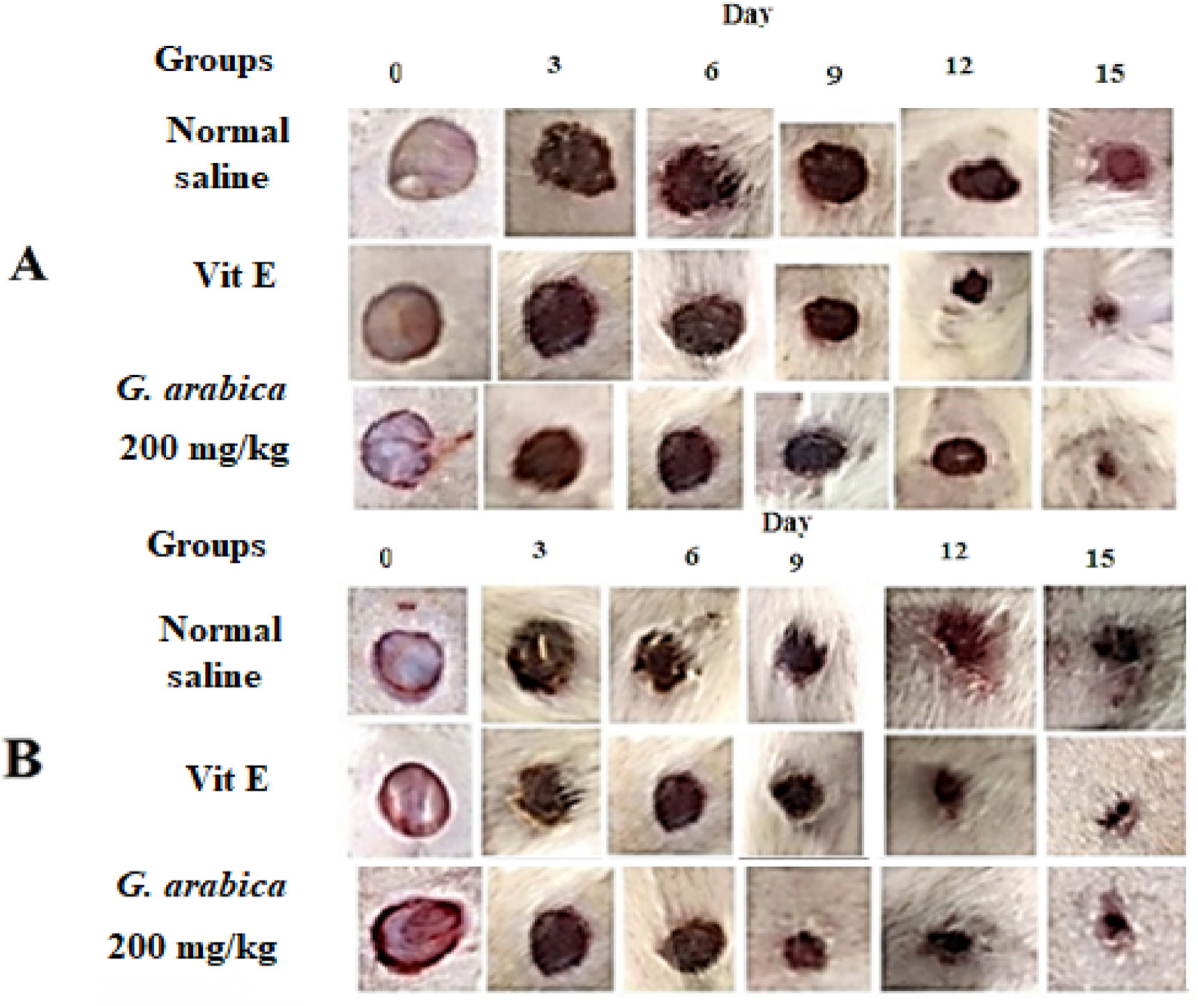
Morphological representations of wound contraction treated orally with *G. arabica* methanolic extracts in non-diabetic (A) and diabetic (B) rats.

The *R. coriaria* methanolic extract (200 mg/kg bw) gave significant (P < 0.05) effects on wound healing as matched to the groups that were given vitamin E (Table 3 and Figure 4). On the other hand, the methanolic extract of *M. sylvestris* (200 mg/kg bw) demonstrated greater healing activity than *G. arabica* (200 mg/kg bw). The healing potential of the two plants is illustrated in Tables 5 and 6, and wound healing processes are depicted in Figures 5 and 6, respectively (see Table 4).

**Table 3:** Effect of orally administration of *R. coriaria* methanolic extracts on wound contraction in non-diabetic and diabetic rats. Values are mean ± S.D (n = 5).

Day	Case	Negative control		Vitamin E (100 mg/kg)		*R.coriaria* (200 mg/kg)	
		Wound diameter (mm)	Percentage of wound contraction (%)	Wound diameter (mm)	Percentage of wound contraction (%)	Wound diameter (mm)	Percentage of wound contraction (%)
0	Non diabetic	10.63 ± 0.31	0	10.39 ± 0.20	0	10.46 ± 0.32	0
	Diabetic	10.37 ± 0.23	0	10.28 ± 0.11	0	10.34 ± 0.09	0
3	Non diabetic	10.37 ± 0.29	2.4	9.55 ± 0.30	8	9.48 ± 0.28	9.3
	Diabetic	10.15 ± 0.21	2	9.61 ± 0.18	6.5	9.55 ± 0.15	7.6
6	Non diabetic	9.76 ± 0.40	8.18	8.78 ± 0.33	15.4	8.16 ± 0.25	21.9
	Diabetic	9.26 ± 0.20	107	8.81 ± 0.32	14.2	8.61 ± 0.27	16.7
9	Non diabetic	9.09 ± 0.68	14.4	6.93 ± 0.64	33.3	6.79 ± 0.52	35
	Diabetic	8.24 ± 0.17	20.5	6.96 ± 0.43	32.2	6.71 ± 0.56	35.1
12	Non diabetic	7.46 ± .0.69	29.8	5.14 ± 0.72	50.5	4.84 ± 0.63	53.7
	Diabetic	7.33 ± 0.21	29.3	5.25 ± 0.74	48.9	4.92 ± 0.59	52.4
15	Non diabetic	5.96 ± 0.76	43.9	1.76 ± 0.23∗	83	1.89 ± 0.30∗	81.9
	Diabetic	5.96 ± 0.41	42.5	2.14 ± 0.36∗	79.1	2.54 ± 0.44∗	75.4

∗ Significant at P < 0.05 over the negative control.

**Table 5:** Effect of orally administration of *G. arabica* methanolic extracts on wound contraction in non-diabetic and diabetic rats. Values are mean ± S.D (n = 5).

Day	Case	Negative control		Vitamin E (100 mg/kg)		*G. Arabica* (200 mg/kg)	
		Wound diameter (mm)	Percentage of wound contraction (%)	Wound diameter (mm)	Percentage of wound contraction (%)	Wound diameter (mm)	Percentage of wound contraction (%)
0	Non diabetic	10.57 ± 0.44	0	10.95 ± 0.55	0	10.75 ± 0.69	0
	Diabetic	10.96 ± 0.7	0	10.66 ± 0.41	0	10.81 ± 0.42	0
3	Non diabetic	10.25 ± 0.08	3	9.95 ± 0.49	9.1	9.85 ± 0.30	8.3
	Diabetic	10.83 ± 0.73	1.18	9.93 ± 0.52	6.8	9.88 ± 0.46	8.6
6	Non diabetic	9.42 ± 0.35	10.8	8.28 ± 0.80	24.3	8.56 ± 0.38	20.3
	Diabetic	9.98 ± 0.52	8.9	8.68 ± 0.55	18.6	7.88 ± 0.51	27.1
9	Non diabetic	8.10 ± 0.22	23.3	6.85 ± 1.04	37.4	7.14 ± 0.37	33.5
	Diabetic	8.54 ± 0.42	22	6.60 ± 0.43	38	6.62 ± 0.47	38.7
12	Non diabetic	7.06 ± 0.55	33.2	3.82 ± 0.62	65.1	4.79 ± 0.58	55.4
	Diabetic	7.55 ± 0.4	31.1	4.94 ± 0.8	63.6	4.82 ± 0.33	55.4
15	Non diabetic	5.69 ± 0.33	46	1.75 ± 0.5∗	84	2.53 ± 0.97∗	76.4
	Diabetic	6.04 ± 0.7	44.8	1.93 ± 0.52∗	81.9	3.32 ± 0.34∗	69.2

∗ Significant at P < 0.05 over the negative control.

**Table 6:** Cytotoxicity and IC_50_ of *R. coriaria*, *G. arabica* and *M. sylvestris* methanolic extracts (n = 3).

Plant	IC_50_ (μg/mL)	Cytotoxicity (%)
*R. coriaria*	90 ± 5.6	37 ± 4.8
*G. arabica*	72 ± 3.3	47.3 ± 5.3
*M. sylvestris*	82 ± 4.2	42 ± 4.8

**Table 4:** Effect of orally administration of *M. sylvestris* methanolic extracts on wound contraction in non-diabetic and diabetic rats. Values are mean ± S.D (n = 5).

Day	Case	Negative control		Vitamin E (100 mg/kg)		*M. sylvestris* (200 mg/kg)	
		Wound diameter (mm)	Percentage of wound contraction (%)	Wound diameter (mm)	Percentage of wound contraction (%)	Wound diameter (mm)	Percentage of wound contraction (%)
0	Non diabetic	10.65 ± 0.56	0	10.82 ± 0.42	0	10.72 ± 0.64	0
	Diabetic	10.63 ± 0.47	0	11.00 ± 0.43	0	11.23 ± 0.53	0
3	Non diabetic	10.45 ± 0.49	1.8	10.18 ± 0.16	5.9	10.05 ± 0.53	6.2
	Diabetic	10.35 ± 0.41	2.6	10.38 ± 0.44	5.6	10.02 ± 0.26	10.7
6	Non diabetic	9.57 ± 0.41	10	8.73 ± 0.53	19.3	8.70 ± 0.42	18.8
	Diabetic	9.40 ± 0.31	11.5	8.60 ± 0.39	21.8	8.18 ± 0.12	27.1
9	Non diabetic	8.57 ± 0.58	19.5	6.26 ± 0.67	42	6.20 ± 0.24	42
	Diabetic	8.21 ± 0.07	22.7	6.31 ± 0.30	42.6	7.42 ± 0.27	33.9
12	Non diabetic	7.54 ± 0.38	29	3.99 ± 0.82	63	5.16 ± 0.11	51.8
	Diabetic	7.29 ± 0.22	31.4	4.43 ± 0.42	59.7	5.30 ± 0.22	52.6
15	Non diabetic	5.79 ± 0.26	45	1.50 ± 0.30∗	86	2.65 ± 0.60∗	75.2
	Diabetic	5.98 ± 0.54	43.7	2.07 ± 0.60∗	81.1	3.18 ± 0.66∗	71.6

∗ Significant at P < 0.05 over the negative control.

Wounds are a clinical problem and they are frequently seen as a serious concern in therapeutic practice. The standard wounds are fixed in a few days, whereas long-lasting wounds are a big problem because of financial and community factors. Because of this, it is important to find new natural products that work better and cost less [42, 43]. Moreover, Diabetes can cause an interruption in the healing process or increase the probability of infection in the wounded area, leading to long hospitalization [44]. Most of the medicines that are previously used for the treatment of wounds are external ointments, however, in this experiment, the plant extracts were administered orally. The present study motivated the improvement of new therapeutic agents using medicinal plant extracts. The results revealed that the untreated group has some degree of wound-healing, which might be due to self-immunity [45], however, the experimental groups that are treated with the methanolic plant extracts showed a faster wound healing process. Moreover, During the wound treatment period, rats treated with methanolic plant extracts exhibited no indications of irritation, discomfort, or scratching at the wound site. This wound-healing activity may occur from the influence of the extracts on the various wound healing phases, such as collagen synthesis, and wound contraction, as previously documented [46]. This interpretation is supported by the present results which displayed that the use of plant extracts leads to a fast wound closure, which causes faster healing. The best results were obtained from *R. coriaria* methanolic fruit extract which may be recommended as another drug for the management of diabetic injuries. It was previously reported that the fruit aqueous extract of *R. coriaria* can cause a decrease in the area of the wounds, increased collagen deposition and hydroxyproline concentration, and reduced collagenase-2 and Myeloperoxidase enzyme activity leading to faster wound healing [47].

In all treated groups, the methanolic extract of *G. arabica* leaves showed a significant (P < 0.05) wound healing efficacy. These results are in accordance with that of Es-Safi et al [48] who reported that the hydroethanolic extract of *G. alypum* leaves possesses a considerable antioxidant agent, mainly due to the presence of flavonoids and phenyl ethanoid constituents. Therefore, these antioxidant compounds may contribute to the healing process. Methanolic extract of *M. sylvestris* leaves exhibited a less healing potential compared to the other two plants. Our results are in agreement with the results of Kovalik et al [49] who reported that *M. sylvestris* leaf extract had no effect on wound healing in the palatal mucosa of rats. In contrast, a previous study done by Pirbalouti et al [50] revealed that the methanolic extract of *M. sylvestris* flowers has a good wound healing activity, and Histological examinations revealed a rise in well-organized collagen bands, an increase in fibroblasts, and a decrease in inflammatory cells. This good healing activity of *M. sylvestris* flower extract could be due to the higher content of anthocyanin, malvin, niacin, and folic acid [50] compared to the *M. sylvestris* leaf extract.

### 3.3 *In vitro* fibroblast proliferation and migration in wound healing

**Figure 7:**
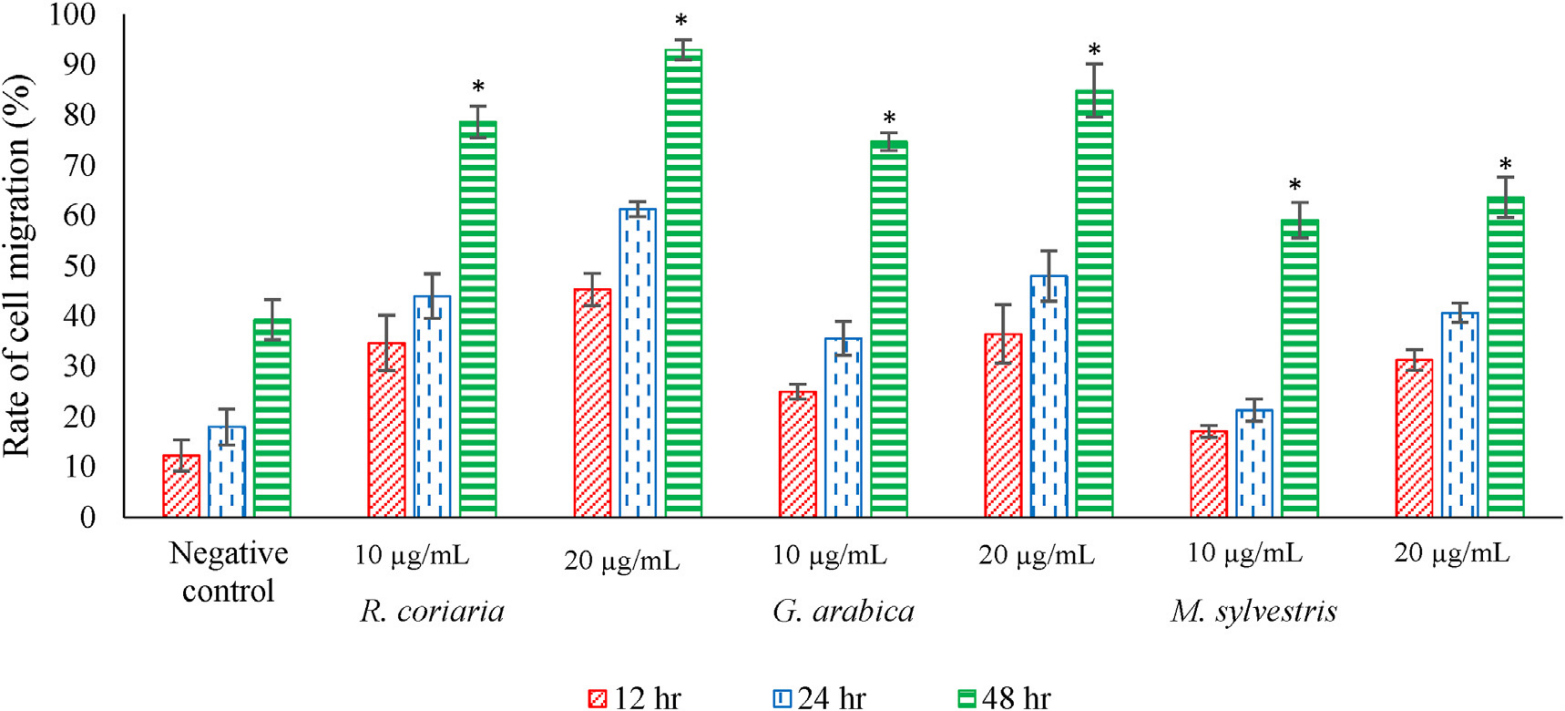
The rate of cell migration of fibroblasts treated with methanolic extracts of *R. coriaria*, *G. arabica* and *M. sylvestris* after 12 h, 24 h, and 48 h compared to the negative control group. ∗ Significant at P < 0.05 over the negative control.

The results of IC_50_ determination and cytotoxicity of plant extracts at a concentration of 100 μg/ml are shown in Table 6. The lowest IC_50_ was seen in *G. arabica*, while the lowest cytotoxicity was seen in *R. coriaria*. The ability of plant extracts to enhance fibroblast proliferation and migration was studied by the scratch method which showed the migration of fibroblasts cell line after scratch assay and during the culture with 10 μg/mL and 20 μg/mL of plant extracts. The highest stimulatory effect was detected after 48 h of treatment with 20 μg/mL of *R. coriaria* extract, followed by *G. arabica* (Figure 7).

Cell proliferation and migration have an important role in the healing process. The scratch method is commonly used for *in vitro* wound healing studies [40]. The fibroblasts are important for collagen deposition, which is required for tissue healing after damage because collagen is important to give the integrity, strength, and structure necessary to return the anatomy and function of damaged tissues [51]. The fibroblasts' activity during the early proliferative phase is cellular replication and migration. On the 3^rd^ day after the injury, however, the enormous mass of fibroblasts begins to produce and create a high amount of collagen in the injured tissues, which defines the wound healing activity [52]. our results showed that the methanolic extracts of *R. coriaria* and *G. arabica* strongly stimulated the migration of the fibroblasts. These results support and confirm the present *in vivo* observations, where the methanolic extract of *R. coriaria* and *G. arabica* showed strong stimulation in fibroblast migration. These results are in agreement with that of Abdallah et al [53] and El Hasasna et al [54], who reported that *R. coriaria* methanolic fruit extract increased uterus cervix cell migration capacity and inhibited cervical and breast cancer growth. Therefore, the present study might find the pharmacological efficiency of the above plants in the healing process.

## 4 Conclusion

This study focused on the pharmacological benefits of the phytotherapy potential of commonly used plants *G. arabica*, *R. coriaria,* and *M. sylvestris*. The significance of this study lies in establishing and reporting for the first time the wound-healing benefits of these plants in a model of diabetic rats. Both the methanolic extracts of *G. arabica* and *R. coriaria* exhibited potent wound healing properties. In addition, the extracts were safe and there were no indications of systemic toxicity in the rats.

## Declarations

### Author contribution statement

Ahmad Z. Alsarayreh, Sawsan A. Oran, Jumah M. Shakhanbeh: Conceived and designed the experiments; Performed the experiments; Analyzed and interpreted the data; Contributed reagents, materials, analysis tools or data; Wrote the paper.

Khaled M. Khleifat, Yaseen T. Al Qaisi, Ibrahim I. Alfarrayeh, Ayah M. Alkaramseh: Performed the experiments; Analyzed and interpreted the data; Wrote the paper.

### Funding statement

This research did not receive any specific grant from funding agencies in the public, commercial, or not-for-profit sectors.

### Data availability statement

Data will be made available on request.

### Declaration of interest's statement

The authors declare no conflict of interest.

### Additional information

No additional information is available for this paper.
